# The Use of Infrared Thermography as a Rapid, Quantitative, and Noninvasive Method for Evaluation of Inflammation Response in Different Anatomical Regions of Rats

**DOI:** 10.1155/2015/972535

**Published:** 2015-03-05

**Authors:** Ireneusz Całkosiński, Maciej Dobrzyński, Joanna Rosińczuk, Krzysztof Dudek, Aleksander Chrószcz, Katarzyna Fita, Robert Dymarek

**Affiliations:** ^1^Department of Nervous System Diseases, The Faculty of Health Science, Wroclaw Medical University, 5 Bartla Street, 51-618 Wroclaw, Poland; ^2^Department of Conservative Dentistry and Pedodontics, The Faculty of Dentistry, Wroclaw Medical University, 26 Krakowska Street, 50-425 Wroclaw, Poland; ^3^Institute of Machines Design and Operation, Technical University of Wrocław, 7/9 Łukasiewicza Street, 50-371 Wroclaw, Poland; ^4^Department of Animal Physiology and Biostructure, The Faculty of Veterinary Medicine, Wroclaw University of Environmental and Life Sciences, 1/3 Kożuchowska Street, 51-631 Wroclaw, Poland

## Abstract

*Purpose*. Thermographic assessment of temperature distribution within the examined tissues allows a quick, noncontact, noninvasive measurement of their temperature. The aim of the study was to evaluate the usefulness of digital infrared imaging in monitoring experimental inflammation of pleura (PL), lower lip (LL), and left paw (LP) and right paw (RP) of lower limbs in rats. *Materials and Methods*. The inflammatory reaction was induced by injection of 1% carrageenin solution into pleural cavity, lip, or paws. With the use of digital infrared imaging temperature measurement was conducted at 0 to 72 hours of the inflammatory reaction. *Results*. The temperature decrease was observed at the site of injection directly afterwards. Next, it was gradually increasing and it reached the maximum on the third day of the inflammatory reaction. Statistically significant changes were observed after 48-hour period in PL and LL regions, as well as after 72-hour period in LP and RP regions (*P* < 0.005). *Conclusion*. It was found that thermographic examination allows for indicating the presence of inflammatory reaction within examined tissues and determining the dynamics of this process. This method could be used as alternative procedure that allows using fewer animals for experiments.

## 1. Introduction

Thermography based on measurements of the level of infrared emission of the examined body surface is one of diagnostic noninvasive methods characterized by large sensitivity [1–3]. Thermography enables registration of the trophic conditions of tissues, as well as thermoregulatory mechanisms, altered areas of tissue metabolism, and inflammatory response [4, 5]. Increased blood flow accompanying inflammatory reaction, as well as increased tissue catabolism, causes a significant rise in local temperature. A map of body temperature is presented graphically and for each temperature scale there is a different colour assigned. A significant and positive feature of diagnostic thermographic measurements is a brief assessment of blood supply of a particular area which proportionally reflects a local rise or drop in temperature, which is directly connected with a series of both functional (i.e., effort hyperemia) and pathological processes, which accompany some phases of the inflammatory reaction. It should be emphasized that there are confirmed difficulties in diagnostics with biochemical examination in the very first stage of inflammation reaction, which is directly related to the local (regional) changes at first, and then it can be observed at the systemic (global) level [6, 7]. By using this method in this research the dynamics of induced local inflammatory reactions along with emphasis of stages of this process connected with local blood flow and metabolic processes of tissues included in the inflammatory process were determined.

It was claimed that thermal imaging methods could not be used to discriminate between procedures and injury inducing an inflammatory response [8]. Nevertheless, thermography was used to observe inflammation reaction caused by contaminated growth promotant ear implants in cattle [9]. Using digital infrared imaging in people allowed diagnosing such changes as inflammatory lesions leading to chronic periodontitis, lingual abscess, salivary gland mixed tumour, or chronic maxillary sinusitis [10]. It is important that thermographic research provided an opportunity to discover subclinical inflammations in horse limbs 14 days before occurrence of clinical symptoms [11]. Digital infrared imaging can be also used as neuroimaging method for displaying cortical temperature in cats, in diagnostics of spine ailments such as microfracture of vertebral bodies, inflammatory states of spinous processes, and intervertebral joint degeneration [12, 13].

At the beginning of inflammatory reaction, the phase of reflectoric contraction of local blood vessels occurs, connected with neurogenic response to the activated pain receptors. Stimulation of those receptors triggers the somatic-vegetative reflex of the adrenal gland [14–16]. After the period of reduced blood supply, there is a local decrease of vascular resistance, which results from secretion of histamine and kinins at further stage. In inflammatory reactions liver metabolism plays an important role as a source of acute phase proteins and clotting cascade proteins, increased proteolysis of muscle proteins, and fever [17–19]. The mentioned mediators cause, as early as within the first hour from the stimulus, vascular reaction in the form of the increase of permeability of vessel endothelium and water shift from plasma to perivascular space. This causes difficulties in outflow of capillary blood from inflammatory focus, which is manifested in oedema and reddening. Despite passive hyperemia in the focus centre hypoxia occurs as a result of local disorders in blood and lymph flow [20, 21]. Febrile reaction is another effect of generalized processes in inflammation. It is caused by secretion of endogenic pyrogens, such as IL-1 and TNF, which are secreted by leucocytic forms and excitation of sympathetic system that accompanies this process (catecholaminemia) and increase of adrenal glucocorticoids [15, 22, 23]. The above described mechanisms of inflammatory reaction connected with changes in blood supply of inflammatory site as well as change of its metabolism cause local increase of temperature independent of febrile state. Changes in temperature distribution may be evaluated with the use of thermography.

Various methods of causing and evaluating experimental inflammatory reaction were described, for example, experimental inflammation with the use of physical factors [24, 25], thermal stimuli, and UV ray [26]. The group of mechanical factors also includes a test connected with implantation of cotton discus which, by irritating nearby tissues, contributes to formation of inflammatory granulation [27]. Most common experimental models of inflammatory process induced by chemical agents include inflammation caused by turpentine oil, formalin, dextran, 10% suspension of bolus alba, acetic acid, and Freud's adjuvant [16, 28, 29]. The oedema evoked by 1% solution of carrageenin is caused by the mediators of inflammatory process such as histamine, serotonin, interleukins, kinins, and prostaglandin [28, 30]. The model of experimental inflammation caused by carrageenin demonstrates reliable results, which were proved in the previous research [28, 31, 32].

The aim of the presented studies was to evaluate the usefulness of infrared thermography as a method assessing the dynamics of inflammatory response to the same factor in different regions of the body. It may contribute to quick diagnostics of an early stage of inflammatory reaction occurring, for example, after injuries and overloads which allows applying less aggressive anti-inflammatory treatment.

## 2. Materials and Methods

Sixteen rat females from* Buffalo* inbreeding strain (the body mass: 150–160 g, age: 10 weeks) were used in these studies. The age and mass body parameters of these animals had to be similar because the reactivity of some inflammatory factors depends on individual features such as age, sex, or strains (under invariable environmental factors). The rats came from breeding in the Department of Pathomorphology of Wroclaw Medical University. All animals received human care in compliance with the Guide for the Care and Use of Laboratory Animals as published by the National Institutes of Health (NRC Publication in 2011). All experiments were performed in compliance with the guidelines for the experimentation on animals. The study was approved by the Local Ethics Council for Animal Experiments (Permission number: 83/2012). All the rats were kept in the same conditions: they were in polystyrene cages (60 cm × 40 cm × 40 cm) with metal lids (6 animals in each cage). The experiments were carried out in the air-conditioned rooms (ambient temperature 20° ± 1°, relative humidity *ϕ* = 50%). The rats were kept in a light/dark cycle for 12/12 h. They were fed with the standard “Murigran” forage and received water* ad libitum *[33].

Before the temperature in the first group was measured, the areas of right intercostal space at the height of 3–5 ribs in the line of elbow joint were chemically depilated with a depilatory cream (Veet, Reckitt Benckiser, Poland) 2 h before the measurement. The animals were immobilized according to the IACUC (Institutional Animal Care and Use Committee) Guidelines modified to visualize the measurement area to meet the experiment's requirements. The injections were made with 0.5 mm needles. The injection depth was 2-3 mm. The control injection of 0.9% NaCl solution was made for all groups. This injection did not cause changes in temperature of the body surface and therefore the data were not included in the results. The experimental animals were divided into 4 groups of four females each:

- group I, in which experimental pleural inflammatory reaction was evoked by intrapleural administration of 1% carrageenin solution (Sigma-Aldrich, USA) in the volume of 0.15 mL between 5 and 6 intercostal space—the region of temperature measurement PL;
- group II, in which experimental inflammatory reaction was evoked by administration of 1% carrageenin solution (Sigma-Aldrich, USA) in the volume of 0.15 mL to a lower lip from the side of oral vestibule—the region of temperature measurement LL;
- group III, in which experimental inflammatory reaction was evoked by administration of 1% carrageenin solution (Sigma-Aldrich, USA) in the volume of 0.15 mL to a foot of lower right limb (paw pad)—the region of temperature measurement RP;
- group IV, in which experimental inflammatory reaction was evoked by administration of 1% carrageenin solution (Sigma-Aldrich, USA) in the volume of 0.15 mL to a foot of lower left limb (paw pad)—the region of temperature measurement LP.

**Figure 1:**
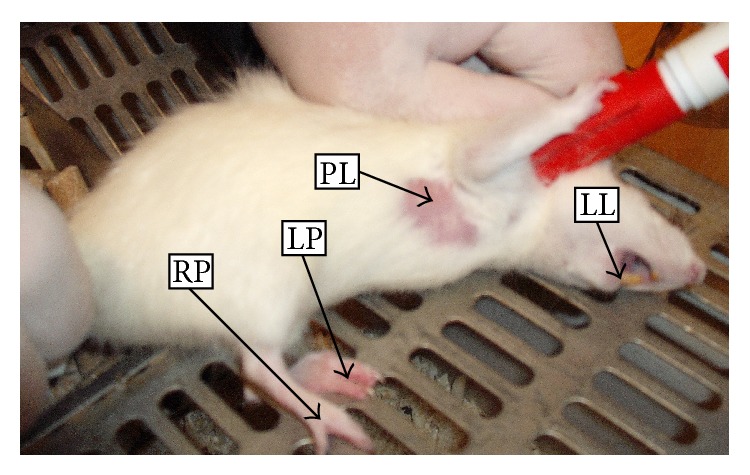
Localization of temperature measurements points in chosen regions of rats: lower lip (LL), pleura (PL), left paw (LP), and right paw (RP).

Localization of temperature measurements points in chosen anatomical regions of rats, lower lip (LL), pleura (PL), left paw (LP), and right paw (RP), is shown in Figure 1. The temperature distribution in the analyzed regions was recorded with the use of digital infrared camera ThermaCAM 550 of the FLIR brand equipped with detector matrix FAT 320 × 240 px of thermal definition < 0.1°C, Stirling cooling system, IR range: 3.6–5.0 *μ*m. To record rat thermograms from a distance of about 20 cm a wide-angle lens and distance ring were used (*T* = 21°C; *ϕ* = 55%). For all analyzed regions (skin) the emissivity *ε* = 0.97 was assumed. The ThermaCAM Researcher Pro 2.8 software was used. Since the subject of analysis was changes in temperature of the skin, any absolute temperature constant error does not significantly affect the results.

The analysis of thermal images consisted in the interactive separation of the analyzed regions of body surface area and determination of quartiles (lower and upper), median, and standard deviation with the use of specialist software of the FLIR brand. Thickness of paws in the examined limb (with inflammatory reaction) and symmetrical side (control) was measured with the use of caliper. Temperature measurements in particular groups, in which experimental inflammatory reaction was evoked within various body regions, were conducted at the following moments:

- control measurement before induction of inflammatory reaction, *t*_0_;
- measurement 10 min after carrageenin administration, *t*_0,2_;
- measurement at 1 h after carrageenin administration, *t*_1_;
- measurement at 2 h after carrageenin administration, *t*_2_;
- measurement at 4 h after carrageenin administration, *t*_4_;
- measurement at 6 h after carrageenin administration, *t*_6_;
- measurement at 12 h after carrageenin administration, *t*_12_;
- measurement at 24 h after inducing inflammatory reaction, *t*_24_;
- measurement at 48 h after inducing inflammatory reaction, *t*_48_;
- measurement at 72 h after inducing inflammatory reaction, *t*_72_;
- measurement at 96 h after inducing inflammatory reaction, *t*_96_;
- measurement at 120 h after inducing inflammatory reaction, *t*_120_.

The animals underwent pharmacological euthanasia with the use of phenobarbital administered intraperitoneally in the amount of 100 mg/kg b.w.

Statistical analysis was performed using the STATISTICA 9.0 software. Temperature distribution in the analyzed sites differed significantly from normal distribution which was verified by the Shapiro-Wilk test at significance level *α* = 0.05. Because of that nonparametric tests were used in the statistical analysis of the temperature measurement results. Statistically significant difference between median temperatures of back paws, examined (with inflammatory reaction) and controlled (without inflammatory reaction), appeared 24 h after a medicine injection. Difference significance was verified by Friedman ANOVA and Kruskal-Wallis ANOVA at the level *P* < 0.05.

## 3. Results

**Figure 2:**
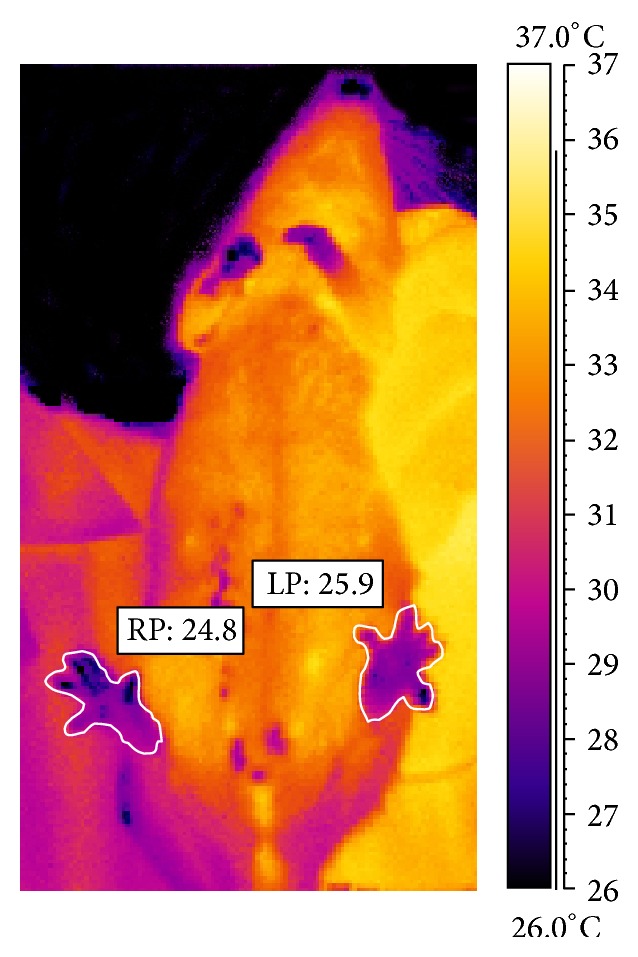
Thermographic imaging showing comparison of mean temperature of rat from group IV made in 24 h after inducing inflammation in left paw (LP) and right paw (RP).

**Figure 3:**
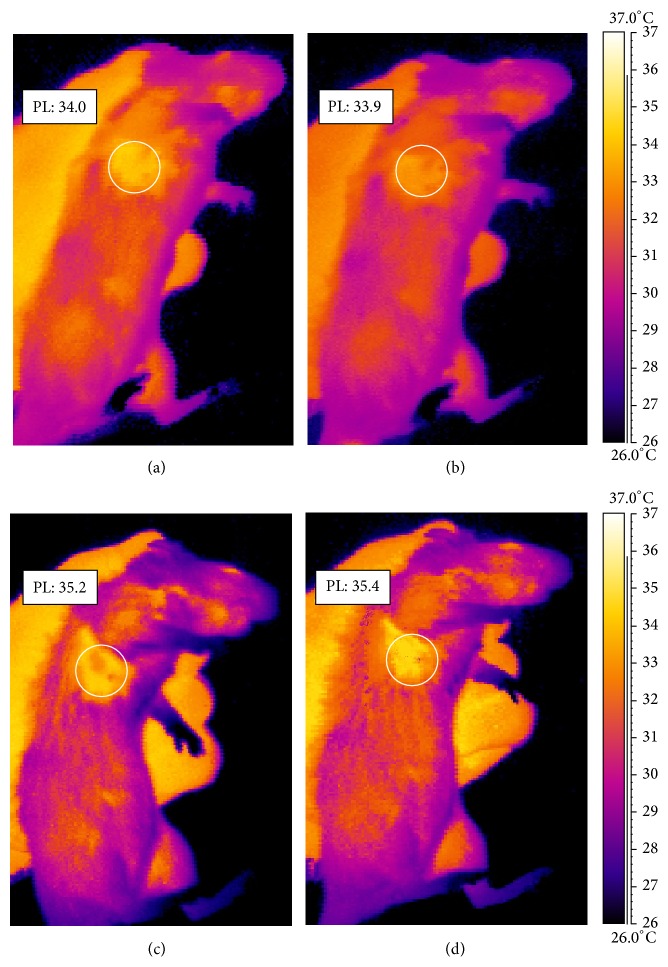
Thermographic imaging showing comparison of mean temperature of rat pleura (PL) from group I made before inducing inflammation (a), 10 minutes after inducing inflammation (b), and 48 (c) and 72 h after inducing inflammation (d).

The thermogram of a rat from group IV (Figure 2) shows the increased temperature of the paws surface with the induced inflammation by 1°C after 24 h after evoking the inflammation. In Figure 3 a thermogram of the course of induced pleural inflammation is presented in which the body surface temperature was growing until 72 h of the inflammatory reaction.

By analyzing temperature measurements of various body regions, on which basis a group classification of experimental animals was made, it has been indicated that the highest temperature is measured in the first group whereas the lowest temperature measurements of the foot area of right and left limbs occurred in groups III and IV. Basic descriptive statistics of temperatures measured with the use of digital infrared imaging are presented in Table 1. The comparison of temperature median distribution for each group shows that the inflammatory reaction dynamics in groups I and II proceeds in a slightly different way compared to groups III and IV where there is a significant body surface in relation to its mass.

**Figure 4:**
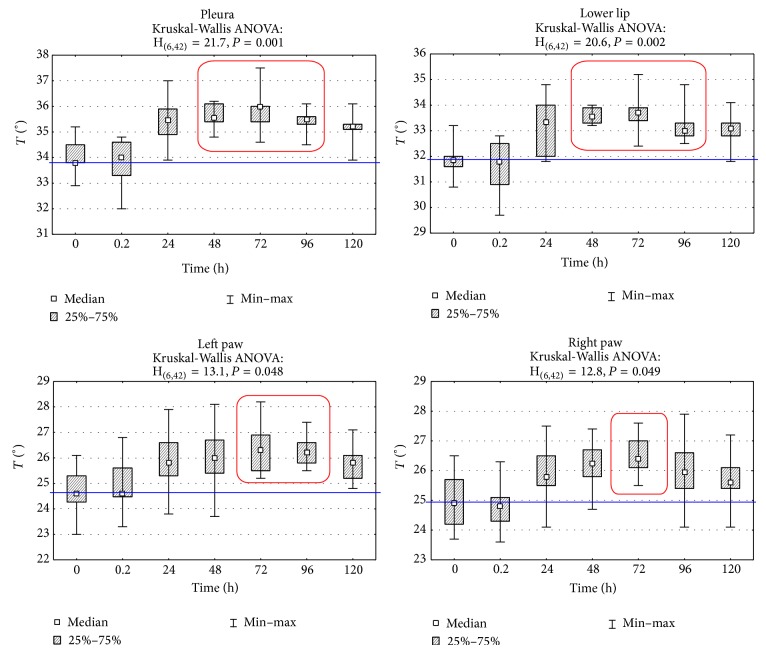
Mean surface temperature of pleura (PL), lower lip (LL), left paw (LP), and right paw (RP) regions in subsequent measurement time points after inducing inflammation. Statistically significant differences are marked with red frames (*P* = 0.001, *P* = 0.002, *P* = 0.048, and *P* = 0.049, resp.)

In group I after 24 h the increase of temperature by 1.7°C was observed. Maximum temperature (increased by 2.2°C) was recorded after 72 h and then a slow drop occurred. However, at 120 h the temperature of the examined area increased by 1.5°C (Figure 4). The dynamics of inflammatory reaction, caused by carrageenin injection into lower lip, revealed maximum increase in temperature equal to 1.9°C compared to control temperature (Figure 4). Observation of the temperature distribution dynamics in the course of experimental inflammatory reaction until 120 h revealed the maximum increase of temperature at 72 h in all groups. The temperature distribution dynamics in groups III and IV was characterized by identical maximum growth occurring between 48 and 72 h of inflammation. The maximum temperature value achieved at that time was 1.3 to 1.5°C higher than control temperature, despite the fact that the increase of maximum temperature during inflammation in those body regions (limbs) was almost twice lower than that in case of induced inflammatory reaction of pleura or lower lip.

**Figure 5:**
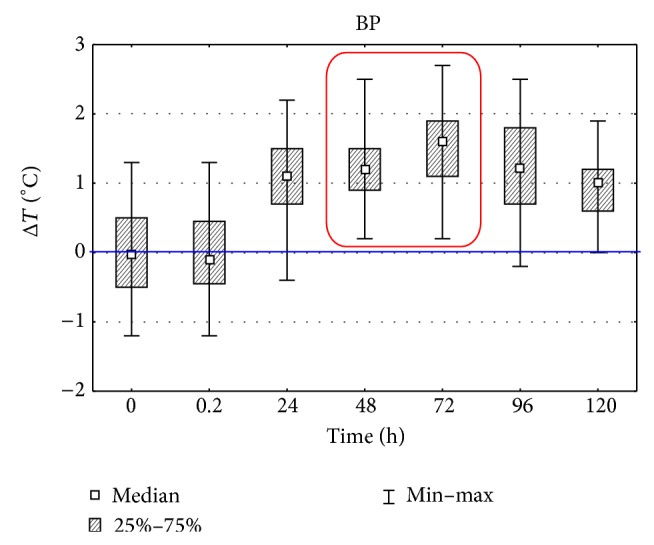
Differences of detected temperature changes of both, studied and symmetric (control), paws (BP) in subsequent measurement time points after inducing inflammation. Statistically significant differences are marked with a red frame (*P* = 0.05).

**Figure 6:**
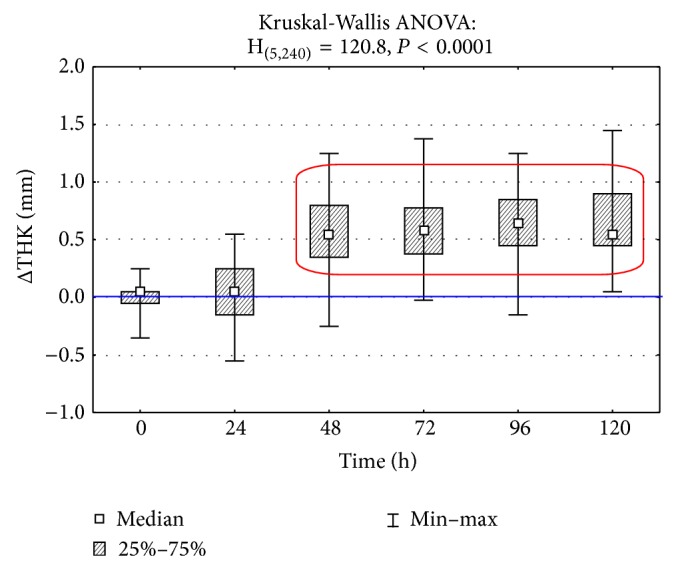
Differences between thickness (THK) of both, studied and symmetric (control), paws (BP) in subsequent measurement time points after inducing inflammation. Statistically significant differences are marked with a red frame (*P* < 0.0001).

In groups III and IV the temperature of a paw surface in which carrageenin was injected and of a paw on symmetrical side without induced inflammatory reaction was measured as well. Differences of temperature between both paws Δ*T* [°C] at consecutive moments are shown in Figure 5. The dynamics of measured temperature differences of foot surface of back left limb and right limb with carrageenin, with reference to the foot of the other limb without carrageenin, revealed the maximum growth between 72 and 96 h since the induced inflammation. In groups III and IV the thickness of paws, examined (with inflammatory reaction) and symmetrical (control), was measured at the same time as temperature with the use of a caliper. A change of thickness difference at other research times is presented in Figure 6. The measure of limb thickness was taken to show oedema, which results from the difficulties in the outflow of capillary blood from inflammatory focus. The obtained temperature measurements correlated with the assessed difference of thickness of back limb feet (Spearman's coefficient *r* = 0.314) where carrageenin was injected in comparison with control limbs without carrageenin, whereas appearance of inflammatory reaction was visible as early as at 24 h of measurement of temperature differences and as late as at 48 h of measurement of differences of paw thickness.

## 4. Discussion

Thermographic evaluation of temperature distribution within the examined tissues may be useful in rapid, noninvasive diagnosis of the inflammatory process. This method also could be applied to assess its initial phase and the acute phase as well as the origin of inflammation. The inflammatory response connected with the histological structure of tissue in the given anatomical area is specific and associated with variability of vasculature and may influence the inflammatory response. A significant element observed in the conducted research is the decrease of local temperature at the site where carrageenin solution was injected 10 min from injection in both the group with induced pleural inflammation and the other with inflammation of right and especially left foot. Hair is effective insulator [34]; therefore it was necessary to remove it to obtain reliable measurement of skin temperature. Hair debris could project a cooler image than is actually present; therefore the hair removing procedure is recommended in thermography studies [8]. According to literature data, the emissivity of the skin ranges from 0.95 to 0.99. Emissivity observed in the temperature ranges from 0°C to 150°C; it does not undergo significant changes, from which this can be assumed as a constant value. In our study we compared the temperature increases, so any error in the determination of the absolute value of the temperature is the systematic error and did not affect the results of the analysis.

The observed temperature decrease within a short period of time since administration of carrageenin solution should be explained by reflex vasoconstrictive reaction connected with excitation of sympathetic system as a result of pain stimulus which is associated with occurrence of somatic-vegetative reflex [35]. It is the first phase of inflammatory reaction connected with sympathetic excitation. It results in local ischemia manifested in local decrease of temperature [28]. Temperature decrease in group I immediately after carrageenin administration may be explained by reflex reflectory contraction of blood vessels connected with reactive superiority of sympathetic system in inflammation just after the stimulus had an effect. It is manifested in the area with a large amount of well blood-supplied tissues. The other effect of the process resulting from ischemia is local hypoxia and the increase of concentration of carbon dioxide and protons associated with it. This leads to release of a series of proinflammatory mediators, that is, histamine, kinin, and prostaglandin [28]. It causes the blood supply increase, which is manifested in the temperature rise until 72 h of the inflammatory process. In that reaction the occurrence of oedema in limbs included in inflammatory process also growing until 72 h which is a follow-up action of secreted proinflammatory mediators may be also observed.

The lowest temperature in groups III and IV was connected with the fact that the measurements concerned peripheral body parts characterized by lesser blood supply as well as great loss of temperature because of a large surface compared to tissue mass. The observed temperature varied with anatomic location and throughout healing, which was also observed in thermography observation of the skin temperature during healing of full-thickness cutaneous wounds created on the horse body and limb [36].

It should be stressed that using digital infrared imaging techniques, apart from observed diagnostic effect, the dynamics of inflammatory process may be investigated. Moreover, it is significant that observations of thermograms from induced inflammatory process coincide, to a large extent, with the increase of concentration of some inflammatory markers whose peak is at 72, 96 h of inflammatory reaction [37].

The authors' observations are similar to those made by Snekhalatha et al. [38] who have shown that during arthritis there is a correlation between thermal imaging measurements in Wistar rats and their ankle diameters. These observations also confirm that thermography could be used to diagnose and analyze inflammatory activity at the preclinical stage. In a number of studies attention is paid to opportunities for using digital infrared imaging in quick diagnostics of hidden inflammatory states before occurrence of clinical symptoms both in people and in animals [12, 39–41]. It was shown that significant thermal changes in the inflammatory reaction related to third molar removal are decreased by the nonsteroidal anti-inflammatory drug [42]. Therefore it is suggested that thermal imaging and oedema measurements could be used for reducing the experimental animals.

Carrageenin injection at the first stage contributed to the decrease of blood supply at the site where the above agent was administered, which resulted in the drop in temperature. In the next phase of inflammatory reaction, at the site of carrageenin injection, the increase in local temperature was observed which reached its maximum at 72 h. It correlates with the observed changes in biochemical blood parameters indicated in our previous studies [33].

## 5. Conclusions 

From the above presented results it may be concluded that infrared thermography can be used as an objective, noninvasive, and quantitative tool determining phases of inflammatory reaction. The presented research results indicate possibility of using digital infrared imaging in quick screening of diagnosis of initiated or hidden inflammatory states. Moreover, while carrying out experiments on animals, application of this diagnostic method reduces the number of animals used in the research because the inflammatory process can be monitored in noninvasive way in not too large group for a long time. The obtained results indicate possibilities of using digital infrared imaging in experimental animals for quick assessment of anti-inflammatory agents activity in pharmacokinetic studies.

## Figures and Tables

**Table 1:** The registered temperature changes in subsequent measurement time points after inducing inflammation in each group.

Measurement time [h]	Group I *T* ^PL^ [°C]	Group II *T* ^LL^ [C°]	Group III *T* ^RP^ [°C]	Group IV *T* ^LP^ [°C]
*t*_0_				
*X* ± SD	34.00 ± 0.59	31.86 ± 0.57	24.66 ± 0.76	24.93 ± 0.85
M	33.8	31.8	24.7	24.9
Min ÷ max	32.9 ÷ 35.2	30.8 ÷ 33.2	23.0 ÷ 26.1	23.7 ÷ 26.5
*t*_0.2_				
*X* ± SD	33.78 ± 0.68	31.64 ± 0.68	24.80 ± 0.71	24.83 ± 0.89
M	34.0	31.8	24.8	24.8
Min ÷ max	32.0 ÷ 34.8	29.7 ÷ 32.8	23.3 ÷ 26.8	22.8 ÷ 26.7
*t*_24_				
*X* ± SD	35.43 ± 0.64	33.30 ± 0.62	25.86 ± 0.98	25.90 ± 0.79
M	35.4	33.3	25.8	25.9
Min ÷ max	33.9 ÷ 37.0	31.8 ÷ 34.8	23.8 ÷ 27.9	24.1 ÷ 27.5
*t*_48_				
*X* ± SD	35.42 ± 0.49	33.26 ± 0.49	26.06 ± 1.03	26.13 ± 0.76
M	35.5	33.3	26.0	26.2
Min ÷ max	33.8 ÷ 36.2	31.5 ÷ 34.0	23.7 ÷ 28.1	23.7 ÷ 27.3
*t*_72_				
*X* ± SD	35.91 ± 0.65	33.73 ± 0.66	26.26 ± 0.98	26.36 ± 0.82
M	36.0	33.8	26.2	26.4
Min ÷ max	34.6 ÷ 37.5	32.4 ÷ 35.2	23.9 ÷ 28.2	24.8 ÷ 27.7
*t*_96_				
*X* ± SD	35.34 ± 0.69	33.19 ± 0.68	26.03 ± 0.96	25.92 ± 0.87
M	35.4	33.2	26.1	25.9
Min ÷ max	33.9 ÷ 36.9	31.8 ÷ 34.8	24.4 ÷ 28.6	24.1 ÷ 27.9
*t*_120_				
*X* ± SD	35.19 ± 0.43	33.04 ± 0.45	25.70 ± 0.69	25.76 ± 0.71
M	35.2	33.0	25.8	25.8
Min ÷ max	34.0 ÷ 36.1	31.8 ÷ 34.1	24.0 ÷ 27.4	24.1 ÷ 27.6

PL: pleura; LL: lower lip; LP: left paw; RP: right paw; *X*: arithmetic mean; SD: standard deviation; M: median value; min: minimal value; max: maximal value.
